# Smartphone-assisted ratiometric fluorescence sensing of hypoxanthine using N, B-co-doped carbon dots for on-site seafood freshness monitoring

**DOI:** 10.1016/j.fochx.2026.104526

**Published:** 2026-09-28

**Authors:** Yaping Zhong, Kayi Shao, Yibiao Zou, Dongmiao Li, Yu Guo, Dong Wang

**Affiliations:** Key Laboratory of Textile Fiber and Products (Wuhan Textile University), Ministry of Education, Hubei International Scientific and Technological Cooperation Base of Intelligent Textile Materials &Application, Wuhan Textile University, Wuhan 430200, China

**Keywords:** Carbon dots, Hypoxanthine, Ratiometric fluorescence, Smartphone detection, Seafood freshness

## Abstract

Hypoxanthine (Hx) is a key biomarker for seafood freshness, accumulating during post-mortem storage. Conventional methods are cumbersome and not suited for on-site use. This work develops a low-cost, portable smartphone-assisted ratiometric fluorescence sensor using N, B-co-doped carbon dots (N, B-CDs) for Hx measurement. An enzymatic cascade yields DAP, quenching the 440 nm blue emission of N, B-CDs and triggering a new orange signal at 570 nm. Glutaraldehyde crosslinks N, B-CDs onto filter paper to fabricate a paper sensor. The liquid-phase assay achieves LODs of 0.106 μM (fluorescence) and 0.072 μM (RGB); the paper sensor enables visual detection from 0.1 to 225 μM with an LOD of 0.128 μM. The platform exhibits ideal selectivity and was validated in fish, shrimp and clam. Freshness classification into fresh, sub-fresh and spoiled batches is realized using R/B ratio thresholds. This work offers a simple, portable and cost-effective tool for on-site seafood quality monitoring.

## Highlights

• N, B-CDs-based ratiometric fluorescent sensor is designed for hypoxanthine detection.
• Cascade enzymatic reaction generates DAP, quenching blue emission of N, B-CDs at 440 nm and emit fluorescence at 570 nm.
• Smartphone-assisted RGB analysis allows visual quantification without instrumentation.
• Chemically grafted paper sensor enables portable, on-site seafood freshness assessment.
• The platform clearly distinguishes fresh, sub-fresh, and spoiled seafood samples.

## 1 Introduction

Seafood, including fish, shrimp, and shellfish, are highly valued worldwide for their excellent nutritional profile, rich in minerals, fatty acids, vitamins and protein (Cao et al., 2024; Che et al., 2025). However, these products are extremely perishable because of their elevated water activity, abundant endogenous autolytic enzymes, neutral pH, and porous tissue structure, which create a favorable environment for microbial growth and biochemical degradation (Wang et al., 2024; Xing et al., 2025). Thus, the timely and precise assessment of seafood freshness is of critical importance for food safety, quality control, and consumer protection. Freshness is a core quality attribute that directly determines the organoleptic characteristics of seafood, such as flavor, texture, smell and appearance (Duan et al., 2022; Hassoun & Karoui, 2015). Among various freshness indicators, key indicators include total volatile basic nitrogen (TVB-N), K-value, total viable count (TVC), and hypoxanthine (Hx). TVB-N mainly reflects the accumulation of alkaline nitrogenous compounds from microbial spoilage, while K-value quantifies the degradation degree of ATP-related purines; however, both indicators require complicated pretreatment or instrumental testing for accurate quantification. As the terminal purine metabolite of adenosine triphosphate (ATP) catabolism, Hx has gained increasing attention as a reliable and stable marker (Dong et al., 2022; Ling et al., 2025; Sun et al., 2025). While the early-stage metabolites (adenosine diphosphate, adenosine monophosphate, inosine monophosphate, inosine) change significantly within the first 24 h and reflect immediate freshness loss, Hx starts to accumulate at the initial storage stage and continues to increase steadily over a longer period (Zhang, Song, et al., 2025). This characteristic makes Hx a more suitable marker for assessing the progressive deterioration of seafood under refrigerated or ambient storage (Gu et al., 2026). Typical Hx levels are below 10 μM in fresh seafood and exceed 50 μM in spoiled products, with species-dependent accumulation kinetics during refrigerated storage. These concentration ranges define the analytical requirements for practical Hx sensors. Consequently, developing a highly responsive, fast, and portable approach for Hx quantification is essential for on-site freshness monitoring.

Conventional methods for Hx detection rely on electrochemical techniques, gas chromatography–mass spectrometry (GC–MS), and high-performance liquid chromatography (HPLC) (Li et al., 2024; Liu, Li, et al., 2024). Although HPLC and GC–MS have favorable analytical reliability and sensitivity, require costly equipment, well-trained personnel, labor-intensive sample preparation, and large volumes of organic solvents, and substantial amounts of organic solvents limits their suitability for fast on-site testing (Fukuuchi et al., 2013; He et al., 2019). Electrochemical methods are relatively simple but often suffer from poor stability in complex food matrices and require frequent electrode modification and calibration (Ibrahim & Temerk, 2016). Thus, there is a pressing need for a simple, cost-effective, and portable analytical platform for real-time Hx detection. Owing to its prominent detection susceptibility, quick response, ease of use, and minimal sample consumption, fluorescence sensing technology has developed into an effective alternative (Liu, Wang, et al., 2024; Zhao et al., 2021). Among various fluorescence strategies, ratiometric fluorescence sensing, which measures the ratio of two emission signals, offers built-in self-calibration against variations in probe concentration, excitation intensity, and environmental factors, thereby significantly improving accuracy and reliability compared to single-intensity measurements. Among various fluorescent materials, carbon quantum dots (CQDs) have drawn tremendous interest due to their tunable fluorescence, excellent photostability, low toxicity, good biocompatibility, and ease of synthesis from abundant precursors (Kang et al., 2025; Zhong et al., 2026). Compared to conventional semiconductor quantum dots, such as CdSe and PbS, CQDs are metal-free and environmentally friendly, making them particularly suitable for food safety applications (Yadav et al., 2023; Zhong et al., 2026). Moreover, heteroatom doping (e.g., with nitrogen, boron, sulfur) can further enhance the fluorescence quantum yield and introduce additional surface functional groups, improving selectivity and sensing performance (Patra et al., 2024; Rishabh, Rani & Shanker, 2026Rishabh, Rani and Shanker, 2026). In particular, nitrogen and boron co-doping provides synergistic effects such as modulating the bandgap and creating active sites, enhancing both fluorescence efficiency and analyte affinity. Paper-based sensors are also attractive for on-site use due to their low cost, disposability, and minimal equipment requirements. Several CQD-based sensors for Hx have been reported recently. For example, Liu et al. proposed Fe, Co, P-co-doped CDs (H. Liu, et al., 2024) and Mou et al. constructed orange-emissive CQDs (Mou et al., 2022). More recently, Liu et al. (Liu, Wang, et al., 2024) reported a physical adsorption-based paper platform using BCNO quantum dots for ratiometric fluorescence sensing of meat freshness. Despite these advances, several critical challenges remain: (1) most reported solid-state sensors rely on physical adsorption, which suffers from limited stability and reproducibility; (2) single-signal readouts cannot provide built-in self-correction; (3) operationally simple visual grading strategies that directly correlate with seafood freshness status and do not require complex computational processing have rarely been demonstrated; (4) most platforms are restricted to either solution or paper formats, without offering complementary detection modes; (5) validation across multiple seafood species with time-dependent spoilage kinetics remains limited.

To overcome these limitations, we herein report a ratiometric fluorescence sensor constructed from nitrogen and boron co-doped carbon quantum dots (N, B-CDs) for the portable detection of Hx (Scheme 1). Our design offers five distinct advantages: (1) covalent immobilization of N,B-CDs onto filter paper via glutaraldehyde crosslinking, overcoming the stability limitations of physical adsorption; (2) the ratiometric readout (F_570_/F_440_) provides built-in self-correction for external disturbances; (3) experimentally validated R/B thresholds (fresh <1.17; sub-fresh 1.17–3.61; spoiled >3.61) enabling immediate visual grading without computation; (4) both solution-based and paper-based formats using the same sensing chemistry; and (5) comprehensive validation on three seafood matrices with time-dependent spoilage monitoring. To our knowledge, this is the first work to integrate covalent grafting, ratiometric readout, validated R/B thresholds, dual-format detection, and multi-species kinetic validation in a single paper-based seafood freshness sensor. The detection mechanism involves the specific enzymatic reaction between Hx and XOD to generate H_2_O_2_, which then reacts with *o*-phenylenediamine (OPD) under catalysis by horseradish peroxidase (HRP) to produce 2,3-diaminophenazine (DAP). DAP efficiently suppresses the blue emission of N, B-CDs at 440 nm through inner filter effect (IFE) and static quenching (SQ), while simultaneously emitting a distinct orange emission peak at 570 nm. The ratio of the two emission signals is directly proportional to Hx concentration, enabling reliable quantification. Furthermore, N, B-CDs was fixed onto filter paper with glutaraldehyde crosslinking to prepare a paper-based sensing platform, which allows visual, instrument-free detection under a UV lamp. The developed sensing platform was effectively used for determining Hx in actual seafood (fish, shrimp, and clam) with ideal selectivity, sensitivity, and recovery. Finally, the primary objectives of this study are: (1) to synthesize N, B-co-doped carbon dots with high quantum yield; (2) to fabricate covalently grafted paper-based sensing strips; (3) to establish validated smartphone-based R/B thresholds for visual freshness grading; (4) to develop both solution-based and paper-based detection formats; and (5) to validate the platform on three seafood matrices with time-dependent spoilage monitoring against HPLC. This work provides a convenient, rapid, and portable platform for on-site assessment of seafood freshness, with great potential for food quality monitoring in resource-limited settings.

**Scheme 1:**
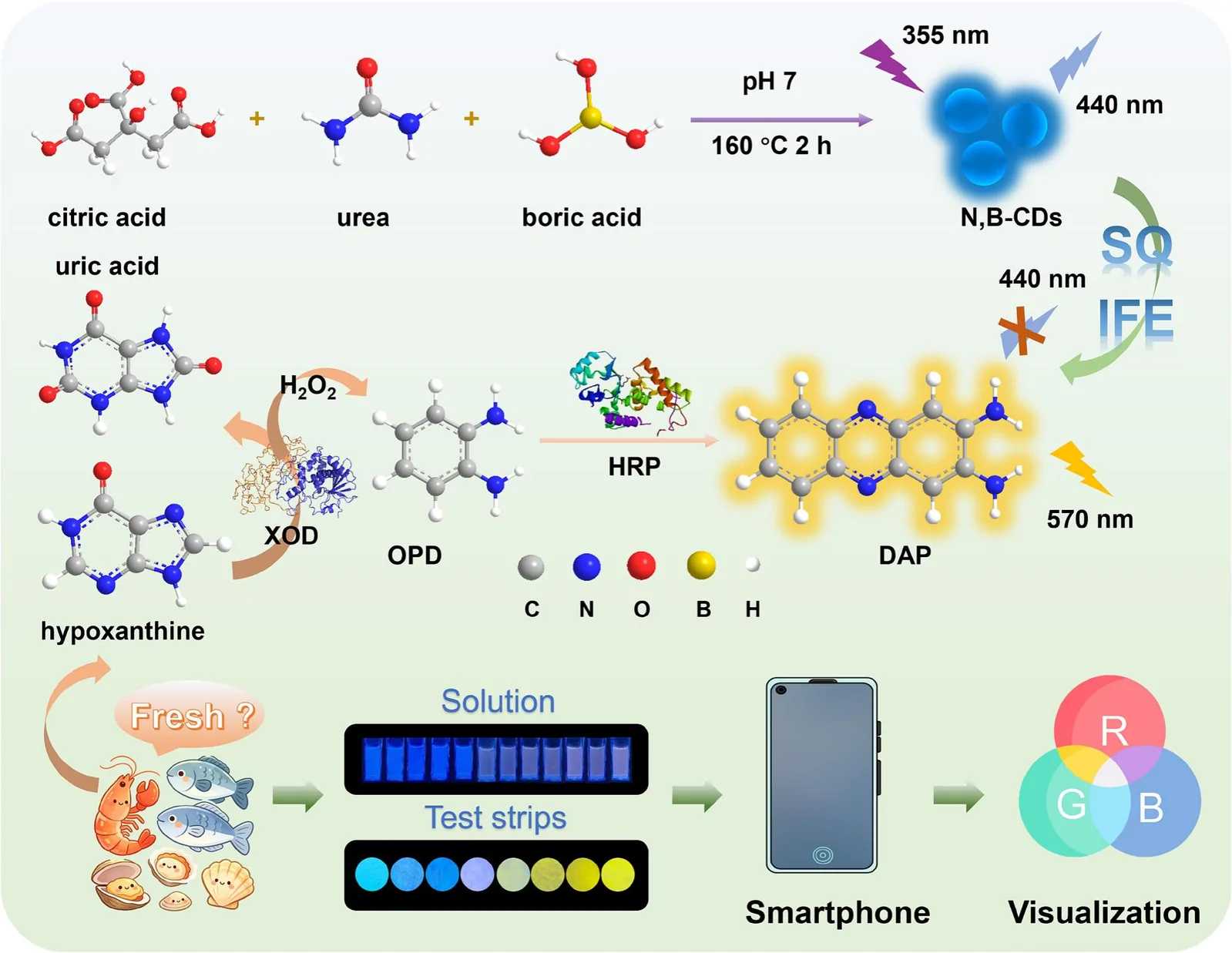
Schematic illustration of the mechanism for hypoxanthine ratiometric fluorescence detection and smartphone assisted on-site seafood freshness evaluation based on N, B-CDs.

## 2 Experimental section

### 2.1 Reagents

Glutathione (GSH, 98%), citric acid (99.5%) and boric acid (99.8%) were obtained from Aladdin Reagent Co., Ltd. Homocysteine (98%), *o*-phenylenediamine (OPD, 99%), lysine (98%), hypoxanthine (Hx, 99%), xanthine oxidase (XOD, 50 U/mg, from bovine milk), aspartate (99%), horseradish peroxidase (HRP, 150 U/mg, from horseradish), alanine (99%), phenylalanine (99%), glutamate (98%), 2,3-diaminophenazine (DAP, 90%) and L-cysteine (99%) were obtained from MACKLIN (Shanghai). Shanghai Sinopharm Chemical Reagents Co. Ltd. supplied Na_2_S_2_O_3_ (99%), MgCl_2_·2H_2_O (98%), NaCl (99.5%), FeCl_3_ (97%), FeSO_4_·7H_2_O (99%), NaHCO_3_ (99.5%), Na_2_CO_3_ (99.8%), CuCl_2_·2H_2_O (99%), CrCl_3_·6H_2_O (99%), KCl (99.5%), CoCl_2_·6H_2_O (99%), NaNO_2_ (99%), NiCl·6H_2_O (98%), MnCl_2_·4H_2_O (99%), ZnSO_4_·7H_2_O (99.5%), KBr (99%), Na_2_SO_4_ (99%), Na_2_SO_3_ (97%), CaCl_2_ (96%), urea (99%) and ascorbic acid (99.7%). All reagents were analytical grade and used as received without further purification. Throughout the experiments, deionized water with a resistivity of 18.2 MΩ·cm was utilized.

### 2.2 Instruments

X-ray photoelectron spectroscopy (XPS) was performed on an AXIS SUPRA spectrometer (Kratos, UK) with monochromatic Al Kα radiation (1486.6 eV); binding energies were calibrated using adventitious C 1 s at 284.8 eV. Transmission electron microscopy (TEM) images were acquired on a JEM-2100 microscope (JEOL, Japan) operated at 200 kV. Fourier-transform infrared (FTIR) spectra were recorded on a VERTEX 70 spectrometer (Bruker, Germany) using KBr pellets. Dynamic light scattering (DLS) measurements were conducted on a ZEN 3600 instrument (Malvern, UK). UV–vis spectroscopy was recorded on a UV-2700 spectrophotometer (Shimadzu, Japan). Solid-state diffuse reflectance spectra were obtained on a Solidspec-3700 spectrophotometer (Shimadzu, Japan) equipped with an integrating sphere attachment; the instrument was baseline-corrected using BaSO_4_ as a reference. Fluorescence spectra were measured on an F97Pro spectrofluorometer (LENGGUANG, China) equipped with a 150 W xenon lamp, with excitation and emission slit widths set to 10 nm; the wavelength calibration was verified using a standard mercury‑argon lamp. X-ray diffraction (XRD) patterns were collected on an Empyrean diffractometer (Malvern Panalytical, Netherlands) using Cu Kα radiation (λ = 1.5406 Å). Fluorescence lifetime measurements were performed on an FLS-1000 spectrometer (Edinburgh Instruments, UK) using a 375 nm pulsed laser diode.

### 2.3 Preparation and fluorescence quantum yield of N, B-CDs and control carbon dots

A one-step hydrothermal strategy was employed to synthesize N, B-CDs. Specifically, citric acid (0.105 g), urea (0.3 g), and boric acid (1.2 g) were dissolved in 20 mL of deionized water. The mixed solution was tuned to neutral pH (7). The mixture was then placed in a Teflon-lined stainless-steel autoclave and maintained 2 h at 160 °C. Following it cooled to room temperature naturally, the product was collected by filtration using a 220 nm microporous membrane. Subsequently, the resulting filtrate underwent dialysis in deionized water with a dialysis membrane (MWCO = 500 Da) over 5 h to acquire purified N, B-CDs suspension, and the final product was preserved at 4 °C for subsequent experiments.

For control experiments, undoped carbon dots (CDs), nitrogen-doped carbon dots (N-CDs), and boron-doped carbon dots (B-CDs) were synthesized under identical conditions (same precursor ratios, temperature, and reaction time) by selectively omitting the corresponding dopants. Specifically, undoped CDs were prepared using citric acid (0.105 g) only, without urea and boric acid. N-CDs were synthesized from citric acid (0.105 g) and urea (0.3 g) without boric acid. B-CDs were synthesized from citric acid (0.105 g) and boric acid (1.2 g) without urea. All control samples were purified by the same dialysis procedure and stored at 4 °C prior to use.

Quantum yield measurement: The relative fluorescence quantum yield (QY) of these carbon dots were determined using quinine sulfate in 0.1 M H_2_SO_4_ (QY = 0.54 at 365 nm excitation) as the reference standard, following the equation: QY_sample_ = QY_ref_ × (F_sample_/F_ref_) × (A_ref_/A_sample_) × (n_sample_^2^/n_ref_^2^), where F is the integrated fluorescence intensity, A is the absorbance at the excitation wavelength (maintained below 0.05 to minimize reabsorption effects), and n is the refractive index of the solvent (1.33 for water and 1.33 for 0.1 M H_2_SO_4_, giving n_sample_^2^/n_ref_^2^ ≈ 1).

### 2.4 Fabrication of paper-based N, B-CDs sensor

Glutaraldehyde was used as the crosslinker in the preparation of the paper-based sensor. Filter paper strips (2 cm × 1.5 cm) were prepared and activated by immersion in 3 mol/L NaOH solution for 30 min. Afterward, the strips were washed with deionized water until neutral and air-drying. The activated paper strips were then immersed in a 2% glutaraldehyde solution prepared in acetate buffer (pH 5.5) and stirred for 30 min. Then washing with deionized water to remove excess glutaraldehyde, the strips were dried. Finally, the glutaraldehyde-treated paper strips were soaked in the N, B-CDs solution with gentle stirring for 2 h. The resulting N, B-CDs-grafted paper strips were washed and dried, yielding the paper-based fluorescence sensor.

### 2.5 Fluorescence detection of Hx in solution

Optimization of analytical parameters. For pH optimization, a series of mixtures containing 20 μL of N, B-CDs, 20 μL of 5 mM Hx, 20 μL of 1 mg/mL XOD, 20 μL of 5 μg/mL HRP, and 20 μL of 5 mM OPD were prepared. Each mixture was then brought to 2 mL with PBS buffers of varying pH values (3–9). After incubation for a fixed time, the fluorescence intensity ratio (F_570_/F_440_) was recorded to identify the optimal pH.

For the time-response study, the same mixture was prepared using PBS buffer at the optimal pH (pH 6), and fluorescence spectra were collected at different time points from 0 to 30 min.

Selectivity study. To assess selectivity, the fluorescence response of the N, B-CDs-based system toward Hx was compared with the responses elicited by various interferents, such as common ions (Zn^2+^, Ni^+^, Cu^2+^, Cr^3+^, Ca^2+^, Co^2+^, Mg^2+^, Fe^2+^, K^+^, Mn^2+^, Fe^3+^, Na^+^, Cl^−^, Br^−^, NO_2_^−^, HCO_3_^−^, CO_3_^2−^, H_2_PO_4_^−^, HPO_4_^2−^, SO_3_^2−^, SO_4_^2−^, S_2_O_3_^2−^), amino acids (aspartate, glutamate, phenylalanine, L-cysteine, lysine, alanine), and other small molecules (citric acid, urea, glutathione, ascorbic acid). Each interferent was tested at the same concentration as Hx, and the fluorescence intensity ratio (F_570_/F_440_) was measured.

Calibration curve. For Hx detection in solution, we mixed different concentrations of Hx (0–250 μM) to 20 μL of N, B-CDs, 20 μL of XOD (1 mg/mL), 20 μL of OPD (5 mM), and 20 μL of HRP (5 μg/mL). The final volume was adjusted to 2 mL using PBS buffer (pH 6). Following 5 min of reaction, emission spectra were recorded. The fluorescence intensity ratio (F_570_/F_440_) was plotted against Hx concentration to obtain the standard working plot. To estimate the limit of detection (LOD), the formula LOD = 3σ/m was applied, with σ defined as the blank standard deviation and m as the slope of the calibration curve from the low-concentration segment. The limit of quantification (LOQ) was similarly calculated using the formula LOQ = 10σ/m, following the IUPAC-recommended criterion.

Additionally, the RGB (red/green/blue) values of the fluorescence color were extracted from photographs taken under a UV lamp. The R/B ratio was plotted against Hx concentration to establish a second calibration curve.

### 2.6 Paper-based detection of Hx

For paper-based detection, all reagents (XOD, HRP, OPD) are not preloaded onto test strips and need to be sequentially dropped onto the sensing zone before measurement. First, 20 μL of Hx solutions at various concentrations (0–250 μM) were dropped onto the N, B-CDs-grafted paper strips and allowed to dry at room temperature. Afterwards, 20 μL of XOD (1 mg/mL), 20 μL of OPD (5 mM), and 20 μL of HRP (5 μg/mL) were sequentially dropped onto the same paper strip. After each reagent dripping, the strip is naturally dried for 1 min at ambient temperature; after all reagents are added, the cascade enzymatic reaction proceeds for 5 min as optimized before UV imaging.

For RGB signal quantification, fluorescence images of the test paper strips were captured using a standardized smartphone-based imaging procedure under a 365 nm UV lamp. The protocol was strictly optimized and fixed to ensure high reproducibility, incorporating the following parameters: a designated smartphone model (Honor 90 Pro) with fixed camera settings (ISO 100, shutter speed 1/30 s, fixed focal length, and auto white balance disabled); a handheld 365 nm UV lamp (6 W) as the excitation source; and fixed imaging distances of 5 cm between the UV lamp surface and the paper strip surface, and 10 cm between the smartphone camera lens and the test strip. All photographing operations were conducted inside a closed dark box to completely eliminate interference from ambient visible light. For RGB data extraction, a commercial Color Recognition software was employed, and a circular region-of-interest (ROI) with a diameter of 6 mm was selected at the center of each fluorescent spot to derive the average R, G, and B channel values. Following the subtraction of background RGB signals obtained from a blank paper area, the background-corrected R/B ratio was calculated for subsequent quantitative analysis.

To address smartphone-to-smartphone variability, we evaluated the paper-based sensor using three different smartphone models: Honor 90 Pro, iPhone 15 Pro, and Huawei Nova 7 5G. The raw R/B ratios for a 50 μM Hx standard showed significant variation (RSD = 9.2%, *n* = 5). A simple normalization strategy using a reference paper strip (N, B-CDs without Hx) with a calibration adjustment factor F_device_ = (R/B_reference,standard_) / (R/B_reference,device_) reduced the inter-device RSD from 9.2% to 3.1% (Honor: 3.21; iPhone: 3.18; Huawei Nova 7 5G), demonstrating satisfactory cross-device compatibility. Ambient lighting (500 lx) increased variability to RSD = 15.4%, confirming the necessity of the dark box environment. We recommend that end-users obtain a reference image of a blank strip using their own smartphone for normalization, eliminating the need for device-specific calibration curves.

### 2.7 Application to real sample analysis

Fresh fish, shrimp, and clam were obtained from a downtown supermarket in Wuhan. To conduct practical sample detection, 2 g of every specimen was blended with 5 mL 10% trichloroacetic acid for homogenization, followed by ultrasonication for 20 min. After centrifugation at 10000 rpm lasting 10 min, the supernatant was harvested and filtrated, with its pH tuned to neutral. The acquired extracting liquid was diluted by 10-fold prior to subsequent quantification. To convert the measured Hx concentration (μM) in the diluted extract to the original tissue content (mg/g wet weight), use the formula:

$$C\;\left(\mathrm{mg}/g\right)=C\;\left(\mathrm{\mu M}\right)\times 10\;\left(\text{dilution factor}\right)\times 0.005\;L\times 136\;g/\mathrm{mol}\times 10^{-6}/2\;g\times 1000$$

Thus, a measured value of 1 μM corresponds to 0.0034 mg/g of wet tissue. All reported tissue concentrations in this work were calculated accordingly.

For solution-based detection, Hx standards were added to the sample extracts to achieve final concentrations of 10 and 70 μM. The fluorescence intensity ratio (F_570_/F_440_) was determined, and Hx levels were calculated using the calibration curve. Recovery experiments were performed to validate the method accuracy.

For paper-based detection, the same sample extracts were dropped onto the N, B-CD-grafted paper strips, followed by the addition of XOD, HRP and OPD solutions. Under UV irradiation, the fluorescence color transition was recorded, and the R/B ratio was measured.

## 3 Results and discussion

### 3.1 Characterization of N, B-CDs

TEM was used to investigate the morphology and microstructure of the prepared N, B-CDs. As shown in Fig. 1a, the N, B-CDs exhibited a spherical morphology and uniformly dispersed with diameter of approximately 2 nm. Fig. S1 shows that the hydrodynamic size is around 10 nm. In the inset of Fig. 1a, the HRTEM lattice spacing of 0.14 nm is consistent with the (100) plane of graphitic carbon. In Fig. S2, a wide peak at around 23.5° (2θ) is observed in the XRD pattern, which assigns to the (002) plane of amorphous carbon, indicating that the N, B-CDs possess low crystallinity and an amorphous structure (Mao et al., 2025). The XPS technique was used to investigate the elemental composition and chemical states of the N, B-CDs. Fig. 1b shows that the XPS survey spectrum of N, B-CDs is dominated by C, N, O, and B elements, with atomic percentages of 51%, 4.68%, 35.37%, and 8.95%, respectively, confirming the successful incorporation of boron and nitrogen dopants into the carbon framework. High-resolution deconvoluted XPS spectra further identify characteristic heteroatom-related bonds: C—B, N—B and O—B bonds from the B 1 s spectrum, as well as C—N and N—B bonds from the N 1 s spectrum. Fig. 1b shows that the XPS survey spectrum of N, B-CDs is dominated by C, N, O, and B elements. High-resolution C 1 s spectrum (Fig. 1c) was separated into four peaks at 287.1, 284.8, 283.9, and 283.1 eV, which are assigned to C=O, C–O/C–N, C—C, and C—B bonds, respectively (Carneiro et al., 2022; Y. Liu et al., 2019). The high-resolution O 1 s spectrum (Fig. 1d) exhibits peaks at 533.9 eV (O—B), 531.1 eV (C—O), and 530.1 eV (C=O) (Zhang, Chen, et al., 2025). The N 1 s spectrum (Fig. 1e) shows three peaks at 400.3, 399.6, and 397.8 eV, corresponding to C—N, N—H, and N—B bonds, respectively (Barman et al., 2024; Chen et al., 2024). The B 1 s spectrum (Fig. 1f) is deconvoluted into three peaks at 191.2 eV (O—B), 190.8 eV (C—B), and 190.3 eV (N—B) (P, K, et al., 2022; Ramajayam & Palaniappan, 2025). The XPS findings above confirm that N, B-CDs were successfully synthesized. The functional groups on the surface of N, B-CDs were identified using FTIR spectroscopy. As shown in Fig. 1g, a broad absorption band in the range of 3500–3000 cm^−1^ corresponds to the O—H and N—H stretching vibrations, indicating the presence of hydroxyl and amino groups. The peak at 1717 cm^−1^ is characteristic of the C=O stretching vibration, while the band at 1075 cm^−1^ is attributed to the C—O stretching vibration. The absorption signal at 1582 cm^−1^ arises from N—H bending vibration, and the peak at 1401 cm^−1^ is attributed to C—N stretching of doped nitrogen heteroatoms, confirming successful N incorporation into the carbon skeleton. Two unique peaks at 1121 cm^−1^ and 981 cm^−1^ are separately indexed to O—B and C—B stretching vibrations, which solidly validate boron co-doping of the carbon dot framework (Chen et al., 2024; Y. Liu et al., 2019). This dual doping introduces additional energy states within the bandgap, which is attributed to the significantly enhanced quantum yield (12.89%) compared to B-CDs (0.17%), N-CDs (1.36%) and undoped CDs (0.35%). Multi-modal characterizations including TEM and XRD describe the morphology and amorphous carbon structure of the carbon-dot host, while XPS and FTIR serve as the direct evidence verifying successful N, B heteroatom co-doping into the carbon framework. As a control, the FTIR spectrum of undoped CDs (Fig. 1g) lacks the characteristic peaks related to N and B, confirming that these peaks arise specifically from N, B heteroatom co-doping. As presented in Fig. 1h, N, B-CDs shows a broad absorption band at 328 nm in the UV–vis spectrum, which is ascribed to the n–π* transition of C=O groups (Ma et al., 2024). The UV–vis absorption spectra (Fig. 1h) also show that undoped CDs exhibit an absorption band at a shorter wavelength, which is blue-shifted compared to N, B-CDs (328 nm), consistent with the electronic effects of heteroatom doping. Using the Tauc equation (*(αhν)*^*2*^ *=* *α*_*0*_*(hν* *−* *E*_*g*_*)*), the optical band gap (E_g_) was determined from the absorption data. From the data in Fig. 1i, the E_g_ value of CDs was calculated to be 5.48 eV, with 5.88 eV for N, B-CDs, both values reflect the strong quantum confinement effect in these small carbon dots (diameter ∼ 2 nm).

**Fig. 1:**
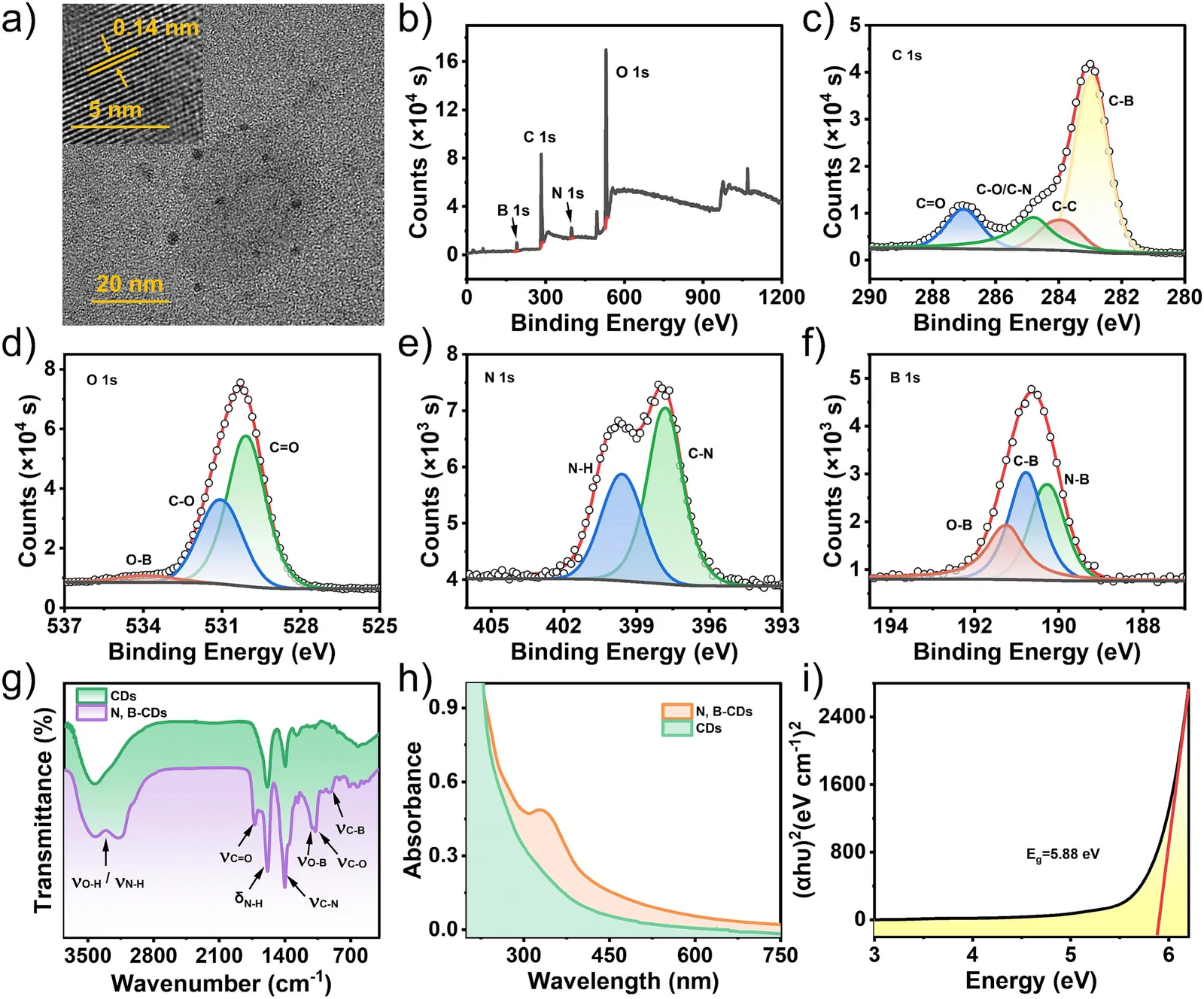
(a) TEM image of N, B-CDs (inset: crystal structure), (b) XPS survey spectrum of N, B-CDs, high-resolution XPS spectra of (c) C 1 s, (d) O 1 s, (e) N 1 s, and (f) B 1 s, (g) FTIR spectrum, (h) UV–vis absorption spectrum of N, B-CDs and CDs, and (i) band gap energy curve of N, B-CDs.

### 3.2 Fluorescence property and stability of N, B-CDs

A systematic study was conducted to evaluate the fluorescence properties of the synthesized N, B-CDs. From the data of Fig. 2a, upon excitation at 355 nm, the N, B-CDs exhibit an intense emission peak centered at 440 nm, indicating the optimal excitation wavelength. Further examination revealed that the N, B-CDs display an excitation-dependent behavior. Specifically, increasing the excitation wavelength from 330 to 380 nm resulted in a gradual shift of the emission peak, as illustrated in Fig. 2b. As the excitation wavelength increases, the CIE chromaticity diagram (Fig. 2c) shows that the emission color gradually changes from blue to greenish-blue. The robustness of N, B-CDs in different environments is critical for their use in food analysis. Therefore, we evaluated the influence of storage time, temperature, ultraviolet (UV) irradiation, ionic strength, and pH on the fluorescence intensity of N, B-CDs. To assess pH stability, the fluorescence intensity of N, B-CDs was measured spanning pH 3 to 9 (Fig. 2d). The results show that the fluorescence intensity exhibited good stability in neutral and alkaline conditions, but decreased slightly under strongly acidic conditions. This pH-dependent behavior is likely due to the protonation or deprotonation of surface functional groups such as carboxyl and amino groups, which can affect non-radiative electron transfer processes. The salt tolerance was examined by adding NaCl at various concentrations of up to 800 mM. As shown in Fig. 2e, only a slight reduction in fluorescence intensity was observed as the NaCl concentration increased. Remarkably, even at 800 mM, the intensity was still above 80% of the initial level, confirming good salt tolerance. This property is particularly beneficial for analyzing real food samples, as they often contain large amounts of salts and other electrolytes. To evaluate the thermal resistance, the N, B-CDs solution was incubated at 4–70 °C for 2 h. The fluorescence intensity remained nearly constant across the entire temperature range (Fig. 2f), demonstrating that N, B-CDs possess ideal thermal stability, allowing it use under various storage and transportation conditions without significant loss of fluorescence. To assess photostability, the N, B-CDs solution was exposed to continuous UV light for 2 h. As shown in Fig. 2g, the fluorescence intensity showed negligible change over this period, indicating strong resistance to photobleaching. Furthermore, the stability under alternating UV light and darkness was tested by cycling between 10 min of UV illumination and 10 min of darkness for a total of 2 h. The fluorescence intensity remained essentially unchanged throughout the cycling experiment (Fig. 2h), confirming that N, B-CDs can withstand intermittent exposure to UV light. This ideal photostability is essential for prolonged detection procedures and for applications that require repeated excitation. Finally, the solution containing N, B-CDs was stored at 4 °C, and its fluorescence intensity was monitored every two days for a total of 30 days to evaluate storage stability. As presented in Fig. 2i, the fluorescence intensity remained almost unchanged during the entire storage period, indicating ideal long-term stability. Collectively, these results demonstrate that the as-prepared N, B-CDs exhibit appealing fluorescence properties and high stability under diverse conditions, suggesting its suitability for practical food quality monitoring applications.

**Fig. 2:**
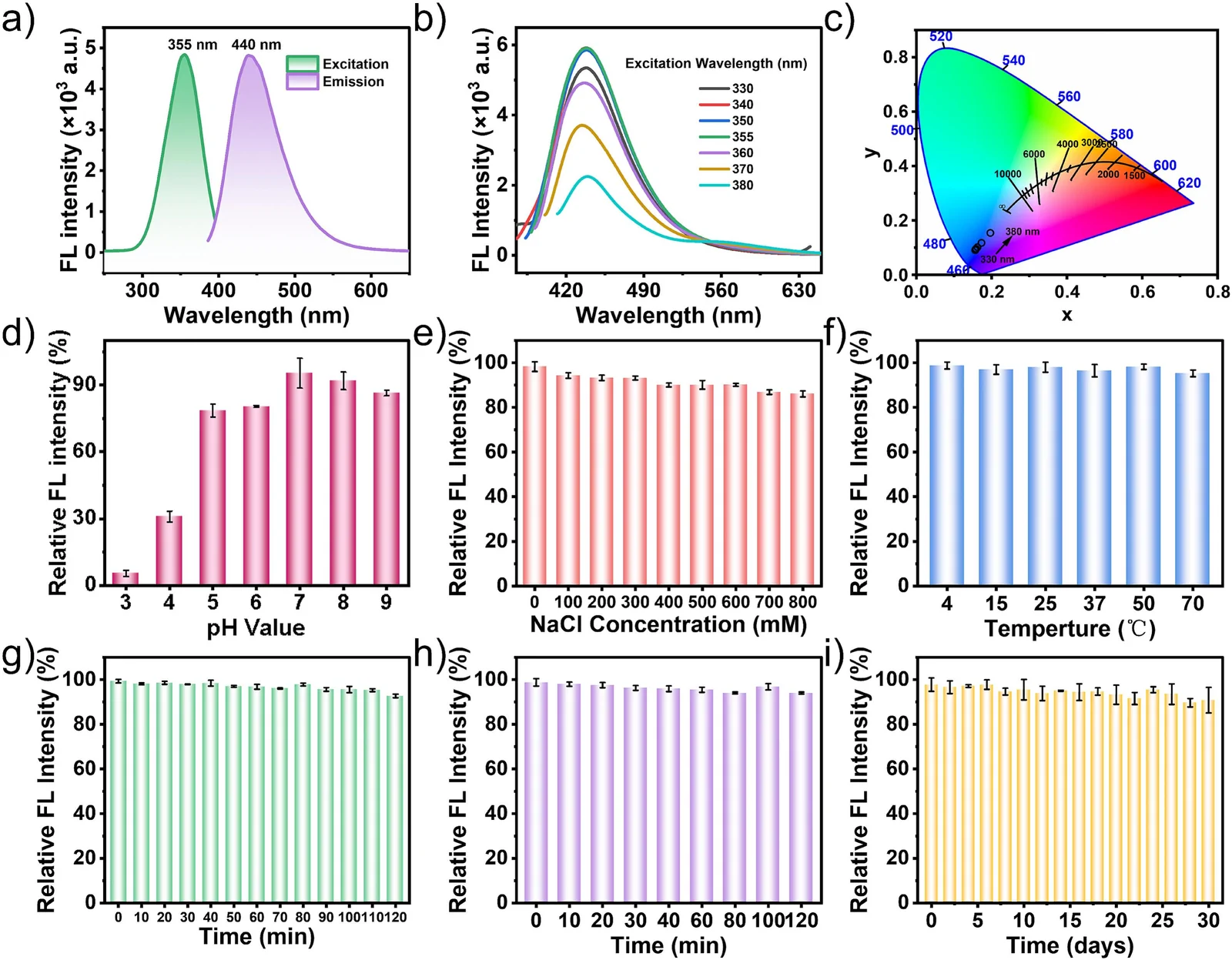
(a) Fluorescence spectrum of N, B-CDs, (b) excitation-dependent fluorescence emission spectra, (c) CIE chromaticity diagram derived from (b), stability of N, B-CDs under (d) different pH values, (e) different salt concentrations, (f) different temperatures, (g) 2 h of UV irradiation, (h) alternating UV irradiation and dark conditions, and (i) different storage times. All data are presented as mean ± SD (*n* = 3 independent measurements per condition). Error bars represent the standard deviation (SD) of three replicates.

### 3.3 Mechanism for the analysis of Hx by N, B-CDs

The detection mechanism of Hx using N, B-CDs is systematically studied. The principle relies on a two-step cascade enzymatic reaction followed by fluorescence quenching. In the first enzymatic step, xanthine oxidase (XOD) serves as the biocatalyst, which selectively oxidizes the target substrate Hx to generate uric acid, with concomitant production of intermediate hydrogen peroxide (H_2_O_2_). In the second enzymatic step, the in-situ-produced H_2_O_2_ then acts as the essential substrate for horseradish peroxidase (HRP), which catalyzes the oxidation of *o*-phenylenediamine (OPD) to produce 2,3-diaminophenazine (DAP) as the key chromophoric and fluorophoric product. Notably, DAP exhibits an absorption band that overlaps with the 440 nm emission of N, B-CDs. Moreover, DAP itself can emit orange fluorescence at 570 nm upon excitation at 440 nm (Fig. S4). Consequently, upon addition of enzymatically generated DAP, the 440 nm emission of N, B-CDs is suppressed by the combined effects of IFE and SQ, where DAP absorbs the fluorescence of N, B-CDs and forms a ground-state complex with them, while a distinct emission peak at 570 nm, assigned to DAP, appears.

To verify this mechanism, control experiments were performed. The fluorescence spectra of these control experiments are summarized in Fig. S4 (with key comparisons highlighted in Fig. 3a and b). As shown in these figures, N, B-CDs alone exhibit strong blue emission at 440 nm, while Hx alone shows no fluorescence. The addition of OPD, HRP, or H_2_O_2_ individually does not affect the fluorescence of N, B-CDs, indicating that these components alone do not quench the N, B-CDs. In the complete enzymatic system without Hx (N, B-CDs + XOD + HRP + OPD), no significant change in the 440 nm emission is observed and no 570 nm peak appears (Fig. 3a and Fig. S4), confirming that DAP is not generated in the absence of Hx. In contrast, the complete sensing system with Hx (N, B-CDs + XOD + Hx + HRP + OPD) shows significant quenching of the 440 nm emission and the appearance of a new 570 nm peak (Fig. 3b and Fig. S4), confirming successful DAP formation and Hx detection. The DAP alone control (Fig. S3) further confirms that the new 570 nm peak originates from DAP. These control experiments collectively verify the proposed sensing mechanism and the essential role of Hx in triggering the cascade reaction. Further mechanistic studies were conducted. The UV–vis absorption spectrum of the Hx + XOD + OPD + HRP reaction mixture showed a new peak at ∼448 nm (Fig. 3c), which is characteristic of the phenazine chromophore in DAP. This absorption arises from the π → π* electronic transition of the extended conjugated system in DAP. The observed red-shift relative to the typical DAP absorption (∼418–430 nm) may be attributed to the protonation of amino groups on DAP under the aqueous buffer conditions (pH 6.0) and the resultant intramolecular charge transfer (ICT) effects. Notably, this peak was absent in control samples without Hx or XOD, confirming that the cascade enzymatic reaction specifically generates DAP only in the presence of Hx. Interestingly, the N, B-CDs + Hx + XOD + OPD + HRP mixture exhibited a newly emerged peak at 404 nm, shifted from the DAP peak at ∼418 nm (Fig. 3e). The absorption intensity at ∼404 nm increased with increasing Hx concentration (Fig. 3d), indicating DAP and N, B-CDs formed a ground-state complex, which is characteristic of SQ. The Stern–Volmer (S—V) plots constructed using the S—V equation (Eq. (1)) at different temperatures further supported the SQ mechanism. As shown in Fig. 3f, the S—V plots exhibited good linearity, and the quenching constant (K_SV_) decreased with increasing temperature, which is typical for SQ. As shown in Fig. 3g, the strong overlap between DAP absorption (418 nm) and N, B-CDs emission (440 nm) further implies that fluorescence quenching is influenced by the IFE or Fluorescence resonance energy transfer (FRET). Fig. 3h shows that the lifetime of N, B-CDs was 6.51 ns, with essentially unchanged to 6.55 ns in the presence of DAP, excluding a FRET contribution. Moreover, the zeta potentials of N, B-CDs and DAP were both negative (Fig. 3i), indicating electrostatic repulsion rather than attraction, further suggesting that the quenching is not due to FRET but IFE.

**Fig. 3:**
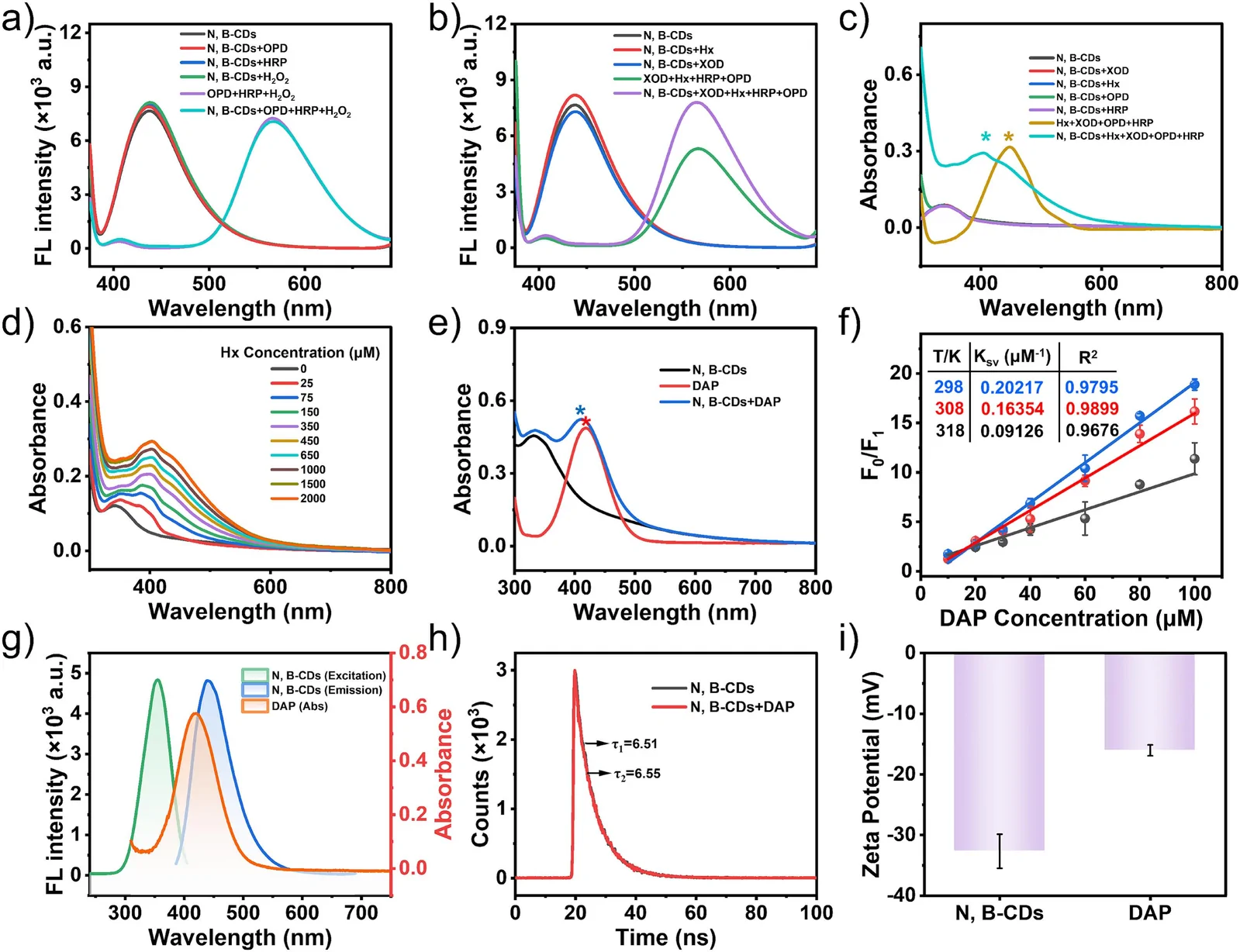
Fluorescence spectra of N, B-CDs and their mixtures in the presence of different substances (a) OPD, HRP, H_2_O_2_, OPD + HRP + H_2_O_2_ and (b) Hx, XOD, Hx + XOD + HRP + OPD, (c) UV–vis absorption spectra of N, B-CDs and their mixtures in the presence of various substances (XOD, Hx, OPD, HRP, Hx + XOD + OPD + HRP), (d) UV–vis absorption spectra of the sensing system at various Hx concentrations, (e) UV–vis absorption spectra of N, B-CDs, DAP, and N, B-CDs + DAP, (f) S—V plots at 25 °C, 35 °C, and 45 °C, (g) fluorescence excitation and emission spectra of N, B-CDs and the UV–vis absorption spectrum of DAP, (h) fluorescence decay curves of N, B-CDs and N, B-CDs + DAP, (i) zeta potential of N, B-CDs and DAP.

In summary, combining the temperature-dependent Stern–Volmer behavior, the unchanged fluorescence lifetime upon DAP addition, and the spectral overlap corrected by absorbance factors, the quenching of N, B-CDs at 440 nm is attributed to the synergistic effect of static quenching (dominant) and inner filter effect (secondary).

Comparative experiments with undoped CDs, N-CDs and B-CDs are summarized in Table S1. Although N-CDs share a similar 440 nm emission band matching the 418 nm absorption of DAP, their low quantum yield fails to generate a high-contrast ratiometric signal. Benefiting from the synergistic effect of N, B co-doping, N, B-CDs maintain the well-matched 440 nm emission and achieve a much higher quantum yield, which endows the ratiometric sensor with superior signal sensitivity.

(1) $$F_{0}/F_{1}=1+K_{\mathrm{sv}}\left[Q\right]$$

Within the equation, F_0_ and F_1_ stand for the fluorescence intensities of N, B-CDs in the absence and presence of DAP, respectively. The symbol [Q] refers to the DAP concentration, whereas K_sv_ denotes the quenching constant.

### 3.4 Hx detection in solution by N, B-CDs

The detection mechanism of Hx using N, B-CDs is schematically illustrated in Fig. 4a. To achieve the best analytical performance, key experimental parameters including pH and reaction time were optimized. The fluorescence intensity ratio (F_570_/F_440_) was used as the response signal to minimize environmental interferences. The optimal pH for Hx detection was determined by measuring the fluorescence intensity ratio (F_570_/F_440_) within the pH span from 3 to 9. Fig. 4b reveals that the maximum fluorescence change was observed at pH 6. This is because, although XOD and HRP are optimally active at pH 7.0–7.5 (Guo et al., 2020; C. Liu et al., 2023) and N, B-CDs fluorescence is highest at pH 7.0 (still ∼80% at pH 6.0 revealed by Fig. 2d), the enhanced inner filter effect from protonated DAP at pH 6.0 compensates for these losses, making pH 6.0 the optimal compromise for the integrated system. Therefore, pH 6 was used in the following experiments. The response time of the sensing system was investigated by monitoring the fluorescence intensity ratio over time after the addition of Hx. Fig. 4c shows that the fluorescence change reached a plateau within 5 min, indicating that the cascade enzymatic reaction and subsequent quenching were completed rapidly. Therefore, all measurements were performed with a reaction time of 5 min to guarantee rapid detection. The fluorescence response of the N, B-CDs-based sensing platform to varying Hx concentrations was investigated under optimal conditions. Fig. 4d presents the fluorescence emission spectra recorded after 5 min of reaction with Hx concentrations (0–250 μM). Upon increasing Hx concentrations, the emission at 440 nm decreased progressively, while the signal at 570 nm showed a corresponding increase. The ratio F_570_/F_440_ was plotted against Hx concentration, yielding two linear ranges (Fig. 4e and f). In the concentration range of 0.1–15 μM, the calibration curve followed the equation Y = 0.11× – 0.0085 (R^2^ = 0.986), while in the 15–125 μM range, it was described by Y = 0.12× + 1.3 (R^2^ = 0.974). The breakpoint at 15 μM was determined by the minimum residual sum of squares method; the two-segment model provides a better fit than a single linear equation across the entire range, which is commonly observed in ratiometric sensors due to the gradual saturation of the quenching effect at higher analyte concentrations. The LOD and LOQ were calculated to be 0.106 μM and 0.353 μM, respectively, which are well below the critical thresholds for freshness loss (10 μM). The CIE curves of the system under various Hx concentrations are shown in Fig. 4g.

**Fig. 4:**
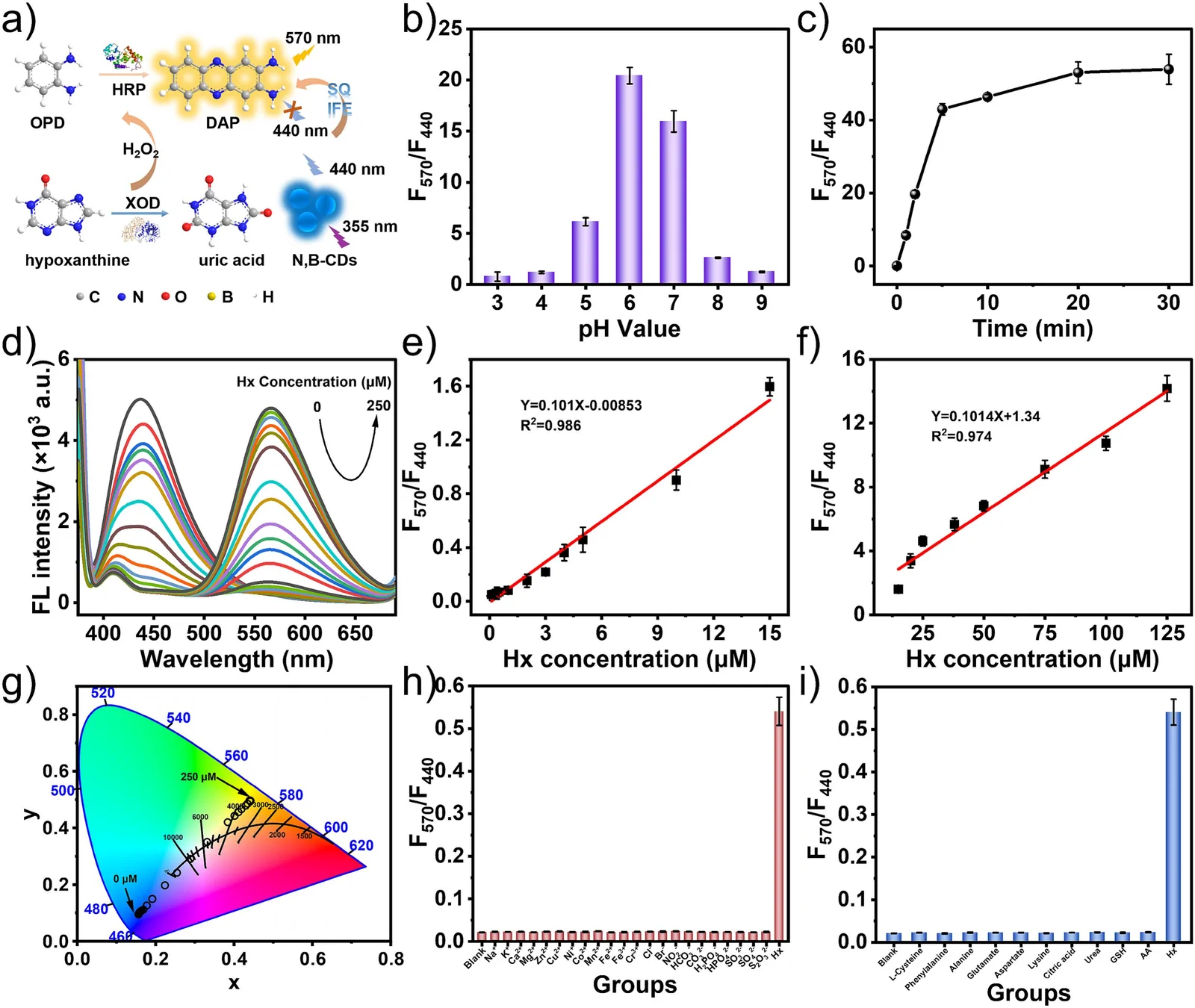
(a) Schematic illustration of N, B-CDs for Hx detection, (b) Fluorescence intensity ratio (F_570_/F_440_) of the sensing system at different pH values (pH 3–9) using 500 μM Hx with a 2 min reaction, (c) Time-dependent F_570_/F_440_ ratio under optimal pH 6 using 500 μM Hx over 0–30 min, (d) Fluorescence emission spectra of the sensing system with varying Hx concentrations (0–250 μM) at pH 6 after 5 min reaction, (e, f) Linear calibration plots of F_570_/F_440_ versus Hx concentration in the ranges of 0.1–15 μM and 15–125 μM, respectively, (g) CIE chromaticity diagram showing the system response to various Hx concentrations based on the F_570_/F_440_ ratio, (h, i) selective detection of Hx.

In addition to fluorescence intensity measurement, we also explored the use of digital color analysis for semi-quantitative visual detection. Under 365 nm UV illumination, the reaction solutions were photographed (Fig. S5a), and the RGB values of each sample were obtained with a smartphone. The CIE chromaticity diagram (Fig. S5b) clearly shows the color evolution from blue (low Hx) to orange-yellow (high Hx), and this change can be directly observed by the naked eye. The relationship between the R/B ratio and Hx concentration was plotted. As shown in Fig. S6a and b, three linear segments were obtained: 0.1–5 μM (Y = 0.072× – 0.0031, R^2^ = 0.996), 5–38 μM (Y = 0.026× – 0.38, R^2^ = 0.973), and 38–225 μM (Y = 0.0059× – 0.47, R^2^ = 0.990). The LOD and LOQ derived from the R/B ratio were 0.072 μM and 0.240 μM, respectively, which are also well below the 10 μM freshness threshold, consistent with the fluorescence measurement results.

Selectivity is a critical parameter for any sensor intended for complex food matrices. We evaluated the fluorescence response of the system toward Hx and a variety of potential interferents, including common ions (Zn^2+^, Ni^+^, Cu^2+^, Cr^3+^, Ca^2+^, Co^2+^, Mg^2+^, Fe^2+^, K^+^, Mn^2+^, Fe^3+^, Na^+^, Cl^−^, Br^−^, NO_2_^−^, HCO_3_^−^, CO_3_^2−^, H_2_PO_4_^−^, HPO_4_^2−^, SO_3_^2−^, SO_4_^2−^, S_2_O_3_^2−^), amino acids (aspartate, glutamate, phenylalanine, L-cysteine, lysine, alanine), and other small molecules (citric acid, urea, glutathione, ascorbic acid). All interferents were tested at the same concentration as Hx. As shown in Fig. 4h and i, a significant rise in the F_570_/F_440_ ratio was observed only for Hx, while the remaining tested substances caused little to no change. This ideal selectivity stems from the specific enzymatic recognition of Hx by XOD. Only Hx can be oxidized by XOD to generate H_2_O_2_, which then triggers the subsequent reactions. Non-specific quenching or interference from other redox-active species was minimal, confirming the method's high selectivity.

### 3.5 Hx detection in real samples by N, B-CDs

The solution-based method was further employed for Hx determination in real seafood samples, including fish, shrimp, and clam, to verify its practical applicability. Table 1 indicated that the Hx concentrations in fish, shrimp, and clam were determined to be 13.34 ± 0.86 μM, 0.72 ± 0.094 μM, and 1.25 ± 0.16 μM, respectively. These values are in close agreement with those obtained by HPLC, demonstrating the satisfactory accuracy of our proposed method. For recovery assessment, Hx standard solutions were introduced into the samples at concentrations of 10 μM and 70 μM. The recoveries ranged from 99.00% to 111.10%, and the relative standard deviations (RSD) were less than 1.10%. The recovery for fish at 10 μM spike was 111.10%, slightly above the acceptable range. To investigate this positive bias, we performed matrix-matched calibration using a blank fish extract to prepare Hx standard solutions. Using matrix-matched calibration, the recovery improved from 111.10% to 105.2% (RSD = 0.8%), confirming that the bias arose primarily from fish matrix effects on the fluorescence signal. This minor bias does not affect freshness classification, as the R/B thresholds (10 μM and 50 μM) are separated by sufficiently large margins. These findings confirm that the proposed solution-based sensor is accurate and reliable for Hx quantification in complex food matrices.

**Table 1:** Fluorescence detection of Hx in fish, shrimp and clam using N, B-CDs in solution (*n* = 3).

Samples	Detected (μM)	HPLC	Spiked (μM)	Found (μM)	Recovery (%)	RSD (%, *n* = 3)
Fish	13.34(±0.86)	14.05(±0.14)	10	24.45(±0.93)	111.10	0.75
			70	83.67(±1.45)	100.47	0.49
Shrimp	0.72(±0.094)	0.68(±0.03)	10	10.62(±0.37)	99.00	0.38
			70	70.89(±0.69)	100.24	0.67
Clam	1.25(±0.16)	1.31(±0.07)	10	11.29(±1.58)	100.40	0.96
			70	71.73(±2.74)	100.69	1.03

### 3.6 Characterization of paper-based sensor

To enable portable and rapid on-site detection, a paper-based sensor was fabricated via glutaraldehyde (GA)-mediated covalent immobilization of N, B-CDs onto cellulose filter paper. The immobilization strategy exploits the abundant amino groups (−NH_2_) on the surface of N, B-CDs and the hydroxyl groups (-OH) on the cellulose paper. The detailed procedure involves three steps. First, filter paper strips were activated by immersion in 3 mol/L NaOH for 30 min to increase the number of surface hydroxyl groups through alkaline hydrolysis of cellulose, enhancing the paper's reactivity. Second, the activated paper was immersed in 2% glutaraldehyde solution in acetate buffer (pH 5.5). Under these conditions, one aldehyde group of glutaraldehyde reacts with the hydroxyl groups of cellulose to form a hemiacetal linkage, leaving the other aldehyde group free for subsequent reaction. The pH 5.5 condition was chosen because it promotes selective hemiacetal formation while minimizing acetal formation or glutaraldehyde self-polymerization. Third, the glutaraldehyde-treated paper was immersed in N, B-CD solution. The free aldehyde groups of the surface-bound glutaraldehyde react with the amino groups on N, B-CDs via Schiff base formation (imine bonds), covalently anchoring the carbon dots onto the paper substrate. The successful immobilization of N, B-CDs onto the paper substrate was confirmed by FTIR, UV–vis, and XPS analyses. A noticeable peak at 1713 cm^−1^ appears in the FTIR spectrum of the N, B-CDs-modified paper (Fig. 5a), which is absent in pristine paper, indicating the C=O stretching vibration of N, B-CDs. Meanwhile, the strong characteristic bands assigned to the C—O and C–O–C stretching vibrations of the cellulose backbone within 1000–1200 cm^−1^ are well retained for the modified paper. Fig. 5b illustrates that the grafted paper exhibits a characteristic UV–vis absorption band at around 328 nm, identical to that of N, B-CDs, confirming retention of their optical properties following immobilization. The XPS survey spectrum of the grafted paper (Fig. 5c) reveals distinct N 1 s and B 1 s signals that are not present in pristine filter paper. High-resolution XPS spectra of C 1 s, O 1 s, N 1 s, and B 1 s for the grafted paper (Fig. 5f–i) further demonstrate the presence of N, B-CDs, with binding energies consistent with those of the N, B-CDs. Collectively, the findings confirm the effective immobilization and stable integration of N, B-CDs on the paper substrate.

**Fig. 5:**
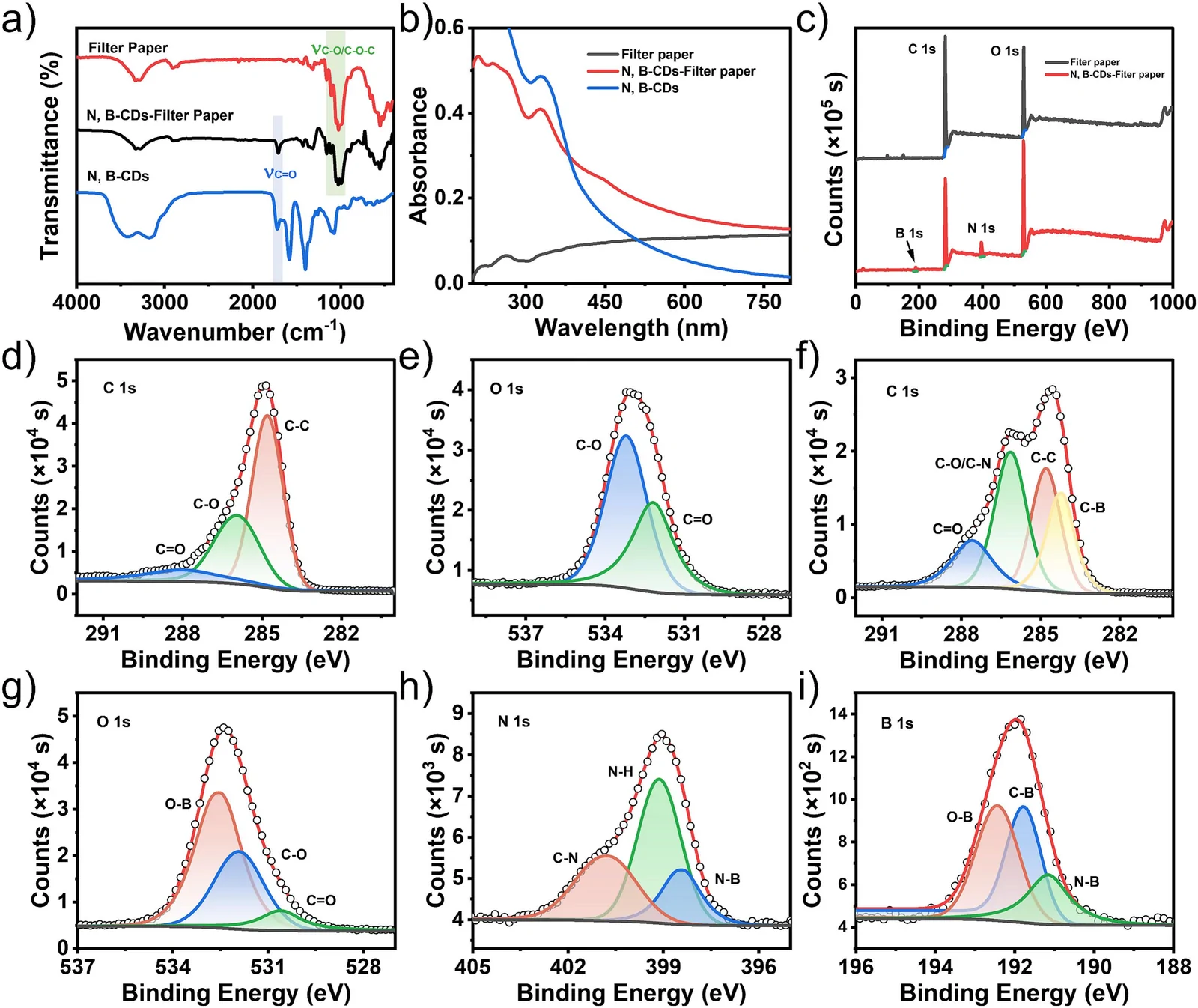
(a) FTIR spectra and (b) UV–vis absorption spectra of filter paper, N, B-CDs-grafted filter paper, and N, B-CDs, (c) XPS full spectra of filter paper and N, B-CDs-grafted filter paper, high-resolution XPS spectra of (d) C 1 s and (e) O 1 s for filter paper, high-resolution XPS spectra of (f) C 1 s, (g) O 1 s, (h) N 1 s and (i) B 1 s for N, B-CDs-grafted filter paper.

### 3.7 Paper-based sensor for Hx detection

The smartphone-assisted paper-based fluorescent sensor shown in Fig. 6a is suitable for Hx detection and determining the freshness of seafood. Different concentrations of Hx solution, together with XOD, OPD, and HRP, were sequentially dropped onto N, B-CDs-grafted paper strips. After 5 min, the paper strips were exposed to 365 nm UV irradiation, and the fluorescence colors were photographed. As shown in Fig. 6b and c, with increasing Hx concentration from 0 to 225 μM, the paper strips displayed a progressive fluorescence color shift from bright blue to orange-yellow. The R/B ratio extracted from the images was plotted against Hx concentration. Four linear ranges were obtained: 0.1–1 μM (Y = 0.69× + 0.070, R^2^ = 0.995), 1–25 μM (Y = 0.061× + 0.56, R^2^ = 0.983), 25–100 μM (Y = 0.024× + 1.9, R^2^ = 0.924), and 100–225 μM (Y = 0.11× – 6.8, R^2^ = 0.978). Breakpoints for piecewise-linear fitting were determined by minimizing residual sum of squares. 95% confidence intervals for slope/intercept and Akaike Information Criterion (AIC)-based model comparison against sigmoidal nonlinear fit are provided in Table S2. The moderate R^2^ of 0.924 for 25–100 μM originates from partial paper-substrate signal saturation; samples falling within this range are recommended for dilution and re-measurement to reduce quantification uncertainty. The sensor exhibited an LOD of 0.128 μM and an LOQ of 0.427 μM, which are comparable to those of the solution-based method and fully adequate for practical freshness assessment. The CIE chromaticity diagram (Fig. S7) illustrates the gradual color transition with increasing Hx concentration. To evaluate selectivity, the paper-based sensing platform was challenged with the same interferents employed in the solution study. As shown in Fig. 6d and e, only Hx produced an obvious shift in color from blue to orange-yellow, while all other tested compounds caused negligible color variations, confirming that the paper-based sensor retains the ideal selectivity of the solution-phase system. The intra-assay repeatability of the paper-based sensing strip was evaluated by measuring the relative R/B ratio of ten independently fabricated identical test strips under uniform conditions (Fig. 6f). Each strip was photographed and analyzed for RGB signals in triplicate to calculate standard deviation, which is presented as error bars in the histogram. All ten parallel strips exhibited highly consistent relative R/B values close to 100%, with negligible deviation within the error bars, demonstrating excellent intra-batch reproducibility and stable signal output of the as-prepared paper sensor.

**Fig. 6:**
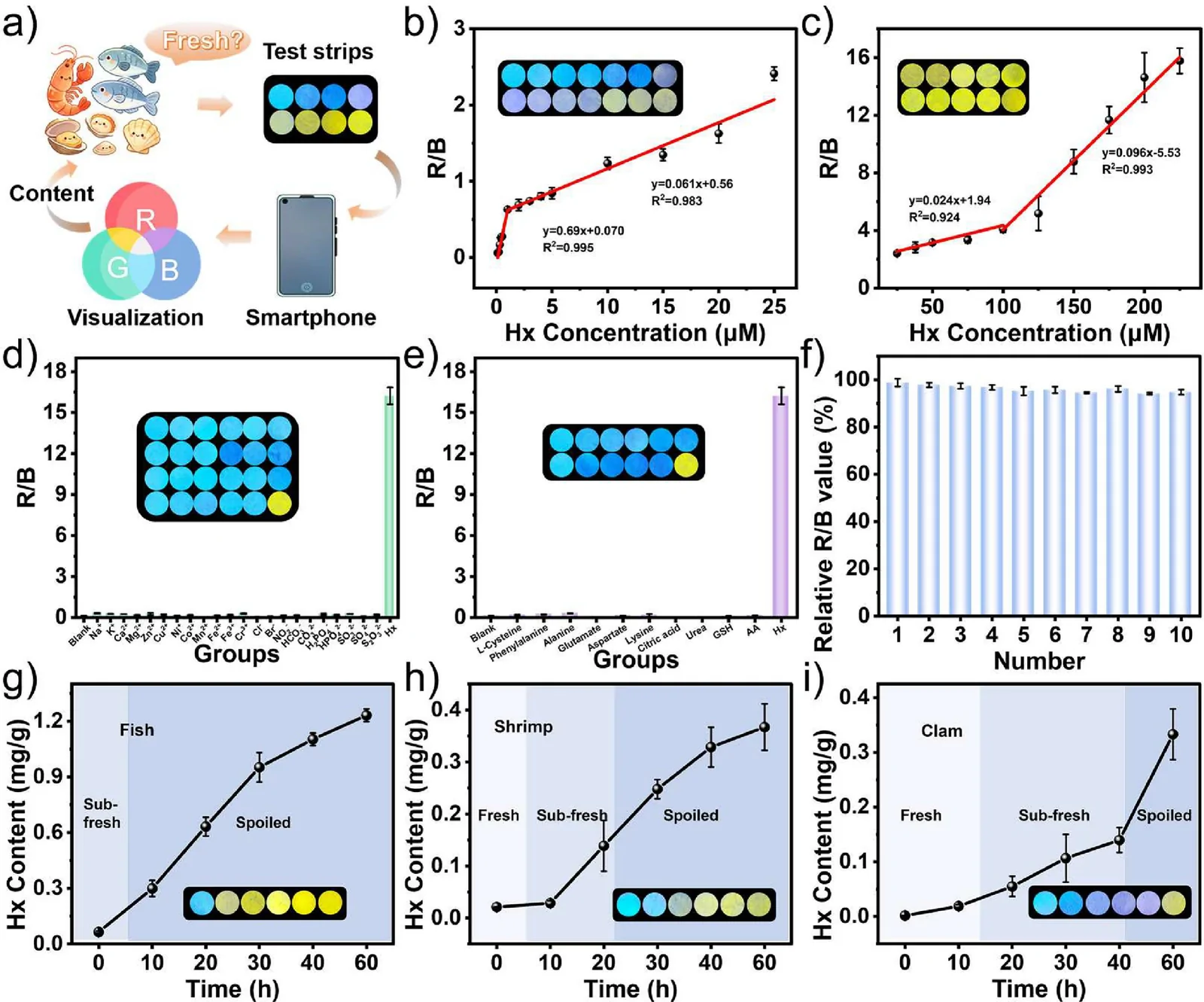
(a) Schematic diagram of the paper-based fluorescent sensing platform for seafood freshness evaluation, linear fitting curves between Hx concentration and fluorescence color R/B value of the paper-based fluorescent sensing platform: (b) 0.1–25 μM and (c) 25–225 μM, (d, e) selective response of the paper-based fluorescent sensor to Hx, (f) intra-batch reproducibility test of the paper-based fluorescent strips, time-dependent changes in Hx content in (g) fish, (h) shrimp and (i) clam samples analyzed by the N, B-CDs paper-based fluorescent sensor.

To translate the measured Hx concentration into a practical freshness evaluation, it is helpful to refer to the generally recognized Hx levels in seafood during storage. According to previous reports, freshly caught or well-preserved aquatic products typically contain Hx below 10 μM, while Hx concentrations exceeding 50 μM are indicative of significant quality deterioration and advanced spoilage (M. Zhang et al., 2025). The linear range of our paper-based sensor (0.1–225 μM) fully covers these critical threshold regions, and the low detection limit (0.128 μM) ensures reliable quantification even at the early stage of freshness loss. Therefore, the paper-based sensor enables a straightforward freshness grading: R/B ratio < 1.17 (blue fluorescence, Hx < 10 μM or < 0.034 mg/g) corresponds to fresh; R/B ratio between 1.17 and 3.61 (blue-green to green, Hx around 10–50 μM or around 0.034–0.17 mg/g) corresponds to sub-fresh; and R/B ratio > 3.61 (orange-yellow, Hx > 50 μM or > 0.17 mg/g) indicates spoiled.

To validate the freshness classification based on R/B thresholds, we analyzed 90 independent seafood samples (30 fish, 30 shrimp, 30 clam) stored at 25 °C for 0–60 h. Each sample was analyzed in parallel by our paper-based sensor and by HPLC as the reference method-. The confusion matrix for the combined dataset (*n* = 90) showed an overall classification accuracy of 91.1% (82/90), with sensitivities of 90.0% for fresh, 86.7% for sub-fresh, and 96.7% for spoiled detection. The R/B ratio correlated well with HPLC-determined Hx concentrations (R^2^ = 0.971, *n* = 90). Notably, no fresh sample was misclassified as spoiled, and no spoiled sample was misclassified as fresh, confirming that the R/B thresholds reliably distinguish the two most practically important categories.

The practical performance of the paper sensing platform was assessed by tracking the freshness of real seafood stored at room temperature (approximately 25 °C) for various storage times (0, 10, 20, 30, 40, and 60 h). For each sampling time, the extract was prepared and introduced onto the paper sensing platform, followed by the XOD, OPD, and HRP solutions. Fluorescence images of the paper strips were captured under UV illumination, and the R/B ratio was calculated. Although the paper substrate exhibits weak autofluorescence, the use of a dark box and background subtraction effectively minimizes its interference, as evidenced by the stable baseline and reproducible R/B ratios in blank controls. The signal-to-noise ratio remains >10 for all tested concentrations, ensuring reliable quantification. As shown in Fig. 6g–i, the R/B ratio increased progressively with storage time for all three samples. This increase directly reflects the gradual accumulation of Hx as the seafood spoiled. Notably, fish exhibited a markedly faster spoilage rate and more rapid Hx accumulation than shrimp and clam under identical ambient storage conditions. The obvious inter-species discrepancy in Hx growth further demonstrates that our smartphone-assisted paper-based sensing platform can realize quantitative freshness grading and differentiate spoilage rates of various seafood matrices for on-site monitoring. The paper sensor was able to clearly distinguish between fresh samples (blue fluorescence) and samples stored for 20 h or longer (orange-yellow fluorescence). This visual, instrument-free readout allows consumers or inspectors to quickly evaluate the freshness status of seafoods without the need for expensive equipment or specialized training.

As shown in Table S3, our platform is distinguished from previously reported systems by several key features: (1) it is the only platform that offers both solution-based and paper-based formats within the same sensing strategy; (2) it is the first paper-based ratiometric Hx sensor that establishes and validates visual freshness classification thresholds, enabling immediate, computation-free freshness assessment; (3) the covalent immobilization strategy provides superior storage stability (>95% after 30 days) compared to most physically adsorbed sensors; (4) the paper-based format requires only 4 operational steps with a 5 min response time, significantly fewer than most electrochemical and HPLC methods; and (5) it is the only system validated on three distinct seafood types with time-dependent spoilage kinetics. These advantages make our method particularly suitable for on-site freshness monitoring in resource-limited settings or at points of sale.

## 4 Conclusions

In summary, a smartphone-assisted ratiometric fluorescent sensing platform using N, B-CDs was constructed for selective and sensitive Hx quantification. The detection mechanism involves the XOD-catalyzed oxidation of Hx to produce H_2_O_2_, which then triggers HRP-catalyzed oxidation of OPD to generate DAP. The blue emission peak of N, B-CDs at 440 nm is efficiently suppressed by DAP through static quenching and inner filter effect, whereas DAP generates orange fluorescence centered at 570 nm, enabling ratiometric readout. Both the solution-based and the paper-based formats exhibit ideal analytical performance, with linear ranges of 0.1–125 μM and 0.1–225 μM, and detection limits of 0.106 μM and 0.128 μM, respectively, fully covering the critical Hx concentration regions relevant to seafood spoilage. Both formats exhibit ideal selectivity against common interferents and satisfactory recoveries (99.00–111.10%, RSD < 1.10%) in fish, shrimp, and clam samples, with results correlating well with HPLC. More importantly, the chemically grafted paper sensor enables a straightforward, visual freshness grading based on experimentally validated R/B ratio thresholds: < 1.17 (fresh), 1.17–3.61 (sub-fresh), and > 3.61 (spoiled), which aligns well with the generally recognized Hx thresholds (10 μM and 50 μM) in aquatic products. Validation against HPLC reference measurements on 90 independent seafood samples gave an overall classification accuracy of 91.1%. Time-dependent spoilage monitoring of seafood confirms that the sensor provides reliable, rapid, and on-site freshness screening. With its low cost, user-friendly operation, and minimal equipment requirements, this platform holds great promise for empowering consumers and inspectors to perform real-time food quality assessment in resource-limited settings.

## CRediT authorship contribution statement

**Yaping Zhong:** Writing – review & editing, Validation, Supervision, Project administration, Investigation, Funding acquisition, Conceptualization. **Kayi Shao:** Writing – original draft, Supervision, Investigation, Formal analysis, Data curation. **Yibiao Zou:** Validation, Supervision. **Dongmiao Li:** Validation. **Yu Guo:** Validation. **Dong Wang:** Validation, Funding acquisition.

## Declaration of competing interest

The authors declare that they have no known competing financial interests or personal relationships that could have appeared to influence the work reported in this paper.

## Data Availability

Data will be made available on request.

## Appendix A Supplementary data

Supplementary material: Hydrodynamic sizes of N, B-CDs; XRD patterns of N, B-CDs; fluorescence emission spectrum of DAP; fluorescence color changes of the system solution at different Hx concentrations; CIE chromaticity diagram of the system based on R/B values in the presence of different concentrations of Hx; linear fitting curves between Hx concentration and fluorescence color R/B values: 0.1–38 μM and 38–225 μM; CIE chromaticity diagram of the paper-based system based on R/B values under varying Hx concentrations; recently reported methods for Hx detection.
