# Supplementary material for: Smartphone-assisted ratiometric fluorescence sensing of hypoxanthine using N, B-co-doped carbon dots for on-site seafood freshness monitoring
Source: Food Chem X. 2026 Sep 28;39:104526. doi: 10.1016/j.fochx.2026.104526 (PMC13651685; doi:10.1016/j.fochx.2026.104526)
Supplement: Supplementary file 1 [file mmc1.docx]

**Smartphone-Assisted Ratiometric Fluorescence Sensing of Hypoxanthine Using N, B-Co-Doped Carbon Dots for On-Site Seafood Freshness Monitoring**

Yaping Zhong^*^, Kayi Shao, Yibiao Zou, Dongmiao Li, Yu Guo, Dong Wang^*^

Key Laboratory of Textile Fiber and Products (Wuhan Textile University), Ministry of Education, Hubei International Scientific and Technological Cooperation Base of Intelligent Textile Materials &Application, Wuhan Textile University, Wuhan 430200, China

*Corresponding author

E-mail addresses:

[ypzhong@wtu.edu.cn](mailto:ypzhong@wtu.edu.cn) (Yaping Zhong);

wangdon08@126.com (Dong Wang).

**Fig. S1.** Hydrodynamic sizes of N, B-CDs.

**Fig. S2.** XRD patterns of N, B-CDs.

**Fig. S3.** Band gap energy curve of CDs.

**Fig. S4.** Fluorescence emission spectrum of different groups.

**Fig. S5.** (a) Fluorescence color changes of the system solution at different Hx concentrations, (b) CIE chromaticity diagram of the system based on R/B values in the presence of different concentrations of Hx.

**Fig. S6.** Linear fitting curves between Hx concentration and fluorescence color R/B values: (a) 0.1–38 μM and (b) 38–225 μM.

**Fig. S7.** CIE chromaticity diagram of the paper-based system based on R/B values under varying Hx concentrations.

**Table S1.** Optical property comparison of different CDs.

| **Different CDs** | **Excitation wavelength (nm)** | **Emission wavelength (nm)** | **QY (%)** |
| --- | --- | --- | --- |
| CDs | 330 | 470 | 0.35 |
| N-CDs | 340 | 440 | 1.36 |
| B-CDs | 400 | 520 | 0.17 |
| **N, B-CDs** | **355** | **440** | **12.89** |

**Table S2.** Statistical parameters for the four-segment piecewise-linear calibration of paper-sensor R/B response.

| **Concentration range (µM)** | **Calibration equation (R^2^)** | **R^2^** | **Slope (95% CI)** | **Intercept (95% CI)** |
| --- | --- | --- | --- | --- |
| 0.1–1 | Y=0.69X+0.070 | 0.995 | 0.69 (0.62, 0.76) | 0.070 (0.041, 0.099) |
| 1–25 | Y=0.061X+0.56 | 0.983 | 0.061 (0.056, 0.066) | 0.56 (0.49, 0.63) |
| 25–100 | Y=0.024X+1.9 | 0.924 | 0.024 (0.019, 0.029) | 1.9 (1.71, 2.09) |
| 100–225 | Y=0.11X−6.8 | 0.978 | 0.11 (0.098, 0.122) | −6.8 (−7.32, −6.28) |

95 % confidence intervals (CI) are shown for slope and intercept parameters. Akaike Information Criterion (AIC) values compare piecewise‑linear fitting versus single sigmoidal nonlinear fitting. Lower AIC value indicates better statistical fitness.

**Table S3.** Recently reported methods for Hx detection.

| **Materials** | **Methods** | **Forms** | **Linear range**  **(µM)** | **LOD**  **(μM)** | **Response**  **Time (min)** | **Operational Steps** | **Enzymes Required** | **Storage Stability** | **Real-Food Validation** | **Visual Freshness Grading** | **Ref** |
| --- | --- | --- | --- | --- | --- | --- | --- | --- | --- | --- | --- |
| MnO_2_-UCNPs | FL | Solution | 3.67-367.35 | 1.03 | 5 | / | Yes (XOD, HRP) | / | Fish | No | [1] |
| MnO_2_ nanosheets | Abs/FL (single emission) | Solution | 5-150/5-125 | 1.506/3.03 | 15 | / | No (nanozyme) | / | Fish | No | [2] |
| Fe_7_Ni_3_MOF | Abs | Solution | 3-70 | 1.39 | 7 | 3 | No (nanozyme) | / | Fish, shrimp | No | [3] |
| Fe, Co, P-CDs | Abs | Solution | 0.3-400 | 0.3 | 20 | 4 | No (nanozyme) | / | Fish, shrimp | No | [4] |
| Pt NPs | FL (single  emission) | Solution | 8-2500 | 2.88 | 30 | 4 | Yes (XOD, HRP) | / | Fish | No | [5] |
| Co(OH)_2_/MXene-Ti_3_C_2_ | Abs | Solution/  Hydrogel | 5-250 | 0.2 | 10 | 3 | No (nanozyme) | / | shrimp | No | [6] |
| 2D Cu-MOF/GN | EC | Solution | 0.01-5 | 0.0052 | / | > 6 | Yes (XOD) | / | Fish | No | [7] |
| 2D Fe MOF | Abs | Solution | 5-500 | 3.29 | 10 | 3 | No (nanozyme) | / | Fish | No | [8] |
| PVP-PtNC | Abs | Solution | 0.5-10000 | 0.16 | 10 | 4 | No (nanozyme) | / | Pork, shrimp | No | [9] |
| AuNPs/GOx/HRP | Abs | Solution | 20-1330 | 20 | 10 | 4 | Yes (GOx, HRP) | / | Fish | No | [10] |
| BCNO QDs | FL (ratiometric) | Solution/  Paper (physical adsorption) | 0-40 (paper) | 0.19 | / | 5 | Yes (GOx, HRP) | / | Pork, chicken | Yes (requires computation) | [11] |
| **N, B-CDs** | **FL (ratiometric)/**  **RGB** | **Solution/**  **Paper (covalent grafting)** | **0.1-125/**  **0.1-225** | **0.106/**  **0.128** | **5** | **4** | **Yes (XOD, HRP)** | **>95% (30 d)** | **Fish, shrimp, clam** | **Yes (R/B thresholds)** | **Our work** |

*Abbreviation: MnO_2_-UCNPs (based on MnO_2_ in-situ coated upconversion nanoparticles), Fe_7_Ni_3_MOF (the nanozyme, derived from the Fe_7_/Ni_3_ metal–organic framework), Fe, Co, P-CDs (Fe, Co, and P codoped CDs), Pt NPs (platinum nanoparticles), Co(OH)_2_/MXene-Ti_3_C_2_ (nanocomposites with OXD-mimic, POD-mimic, and CAT-mimic activities), 2D Cu-MOF/GN (2D copper-based metal-organic framework nanosheets integrated in 2D graphene nanosheets), 2D Fe MOF (two dimensional iron metal-organic framework nanosheet), PVP-PtNC (polyvinylpyrrolidone-modified platinum cubic nanomaterials), AuNPs/GOx/HRP (a freeze–thaw induced co-assembly (FTIA) of multi-enzyme-AuNPs hybrids), BCNO QDs (boron carbon oxynitride quantum dots).

**References**

[1] Y. Cao, Y. Song, T. Wei, T. Feng, M. Li, C. Xue, J. Xu, MnO_2_ in-situ coated upconversion nanosystem for turn-on fluorescence detection of hypoxanthine in aquatic products, Food Chem. 431 (2024) 137131, <http://dx.doi.org/10.1016/j.foodchem.2023.137131>.

[2] M. Zhang, N. Song, X. Wang, J. Chen, J. Choo, X. Wang, L. Chen, The colorimetric and ratiometric fluorescence dual-mode detection of hypoxanthine through catalytic reactions for assessing aquatic product freshness, Food Chem. 475 (2025) 143274, <http://dx.doi.org/10.1016/j.foodchem.2025.143274>.

[3] Z. Li, L. Cao, J. Sui, L. Wang, H. Lin, K. Wang, Bimetallic Fe/Ni metal organic framework-based hypoxanthine biosensor for early monitoring of freshness changes of aquatic products, Food Chem. 447 (2024) 138902, <http://dx.doi.org/10.1016/j.foodchem.2024.138902>.

[4] H. Liu, R. Wang, D. Zhao, D. Liang, C. Zhang, Y. Jiao, X. Xiao, Preparation of Fe,Co,P-Codoping Peroxidase-like Green-Emitting Carbon Dots and Its Application in Monitoring the Freshness of Aquatic Products, J. Agric. Food Chem. (2024) <http://dx.doi.org/10.1021/acs.jafc.4c06379>.

[5] J. Chen, Y. Lu, F. Yan, Y. Wu, D. Huang, Z. Weng, A fluorescent biosensor based on catalytic activity of platinum nanoparticles for freshness evaluation of aquatic products, Food Chem. 310 (2020) 125922, <http://dx.doi.org/10.1016/j.foodchem.2019.125922>.

[6] Q. Yang, Y. Qin, T. Lei, Y. Zhong, X. Cui, Y. He, G. Song, Co(OH)_2_/MXene-Ti_3_C_2_ nanocomposites with triple-enzyme mimic activities as hydrogel sensing platform for on-site detection of hypoxanthine, Microchimica Acta 189 (2022), <http://dx.doi.org/10.1007/s00604-022-05584-z>.

[7] C. Li, Y. Qian, Y. Li, C. Wu, Y. Tang, Unique 2D/2D heterojunctions for assessing freshness and quality of fish products, Microchem. J. 216 (2025) 114673, <http://dx.doi.org/10.1016/j.microc.2025.114673>.

[8] H. Wu, Z. Xu, D. Xiong, X. Qin, G. Liu, H. Zhang, Two dimensional iron metal-organic framework nanosheet with peroxidase-mimicking activity for colorimetric detection of hypoxanthine related to shrimp freshness, Talanta 265 (2023) 124833, <http://dx.doi.org/10.1016/j.talanta.2023.124833>.

[9] X. Ma, T. Feng, P. Zhang, H. Zhang, X. Hu, Y. Yang, Z. Wang, H. Zhang, D. Peng, X. Li, J. Xu, Downregulation of Peroxidase Activity of Platinum Cube Enables Minute–Time Scale Colorimetric Signaling of Hypoxanthine for Fish Freshness Monitoring, Foods 12 (2023) 291, <http://dx.doi.org/10.3390/foods12020291>.

[10] R. Sun, R. Lv, T. Du, Y. Li, Y. Zhang, L. Chen, Y. Qi, Freeze-thaw induced co-assembly of multi-enzyme immobilized AuNPs probes for fast detection of glucose and hypoxanthine, Microchem. J. 181 (2022) 107755, <http://dx.doi.org/10.1016/j.microc.2022.107755>.

[11] X. Liu, Z. Li, Z. Wang, X. Cui, W. Li, M. Wang, Y. He, BCNO quantum dots-based ratiometric fluorescence platform integrated with portable device: Hypoxanthine sensing for on-site assessment of meat freshness with deep learning, Chem. Eng. J. 480 (2024) 147917, <http://dx.doi.org/10.1016/j.cej.2023.147917>.
